# Myxedema-related cardiac tamponade diagnosed by focused cardiac ultrasound (FoCUS): a case report

**DOI:** 10.3389/fcvm.2026.1753361

**Published:** 2026-02-06

**Authors:** H. A. Nati-Castillo, Martin Ocampo-Posada, Wilfredo Antonio Rivera-Martínez, Fredy Lizarazo Davila, Alice Gaibor-Pazmiño, Marlon Rojas-Cadena, Juan S. Izquierdo-Condoy

**Affiliations:** 1 Grupo de Investigación en Biomateriales y Biotecnología - BEO, Facultad de Salud, Universidad Santiago de Cali, Cali, Colombia; 2 Grupo de Investigación en Ciencias Básicas y Clínicas de la Salud, Universidad Javeriana, Cali, Colombia; 3 Endocrinology Department, IPS Nerum, Medellín, Colombia; 4 Interinstitutional Group of Internal Medicine (GIMI I), Universidad Libre, Cali, Colombia; 5 One Health Research Group, Universidad de Las Américas, Quito, Ecuador

**Keywords:** cardiac tamponade, focused cardiac ultrasound, hypothyroidism, myxedema, pericardial effusion

## Abstract

**Introduction:**

Pericardial effusion can progress to cardiac tamponade, a life-threatening condition in which clinical findings are often nonspecific and a complete Beck's triad is frequently absent. Focused cardiac ultrasound (FoCUS) provides rapid bedside recognition of tamponade physiology and can expedite definitive management. This case highlights its pragmatic value for promptly identifying tamponade in a rare endocrine etiology—severe hypothyroidism (myxedema)—particularly in resource-limited settings.

**Case presentation:**

A 62-year-old man with a 6-month history of progressive lower-limb edema and exertional dyspnea deteriorated to dyspnea at rest and generalized anasarca. Examination showed myxedematous features, jugular venous distension, and muffled heart sounds. Bedside FoCUS demonstrated a massive circumferential pericardial effusion (∼28 mm in diastole) with right-atrial systolic and right-ventricular diastolic collapse, consistent with cardiac tamponade. Emergency pericardiocentesis drained 1,200 mL of serous fluid with immediate clinical improvement. Chest radiography obtained before drainage showed a “water-bottle” cardiac silhouette, and pericardial fluid studies were negative for infection and malignancy. Thyroid testing confirmed severe primary hypothyroidism (TSH 225 mIU/L; free T4 < 0.07 ng/dL). The patient was treated with monitored levothyroxine and had no recurrence of pericardial effusion on follow-up.

**Conclusions:**

Severe hypothyroidism is an uncommon but clinically important cause of cardiac tamponade. In patients with unexplained cardiomegaly or hemodynamic compromise, early FoCUS—integrated with clinical assessment and targeted biochemical testing—can rapidly confirm tamponade physiology, accelerate lifesaving pericardiocentesis, and guide timely definitive therapy. FoCUS should be incorporated into routine emergency and critical-care workflows as an extension of the physical examination.

## 1 Introduction

The pericardium, composed of visceral and parietal layers, forms a protective sac around the heart and maintains a potential space with lubricating and insulating functions (1–3). Disorders of the pericardium encompass a wide range of etiologies, including inflammatory, infectious, traumatic, iatrogenic, pharmacological, and systemic conditions (3, 4). While idiopathic pericardial disease predominates in developed countries (3), uremic, infectious, and neoplastic etiologies remain common in many settings; nevertheless, a substantial proportion of cases are ultimately classified as idiopathic (5).

Pericardial effusion is defined as the accumulation of ≥50 mL of fluid within the pericardial cavity. In some cases, the effusion may progress to cardiac tamponade, a life-threatening condition characterized by impaired ventricular filling and reduced cardiac output. Classically, tamponade is suspected when jugular venous distension, hypotension, and muffled heart sounds (Beck's triad) are present; however, this triad is often incomplete or absent, making the clinical diagnosis challenging. Moreover, symptoms such as dyspnea, tachycardia, and peripheral edema frequently overlap with other cardiopulmonary disorders, particularly in early stages (3, 4, 6).

In this context, point-of-care ultrasound (POCUS)—and especially focused cardiac ultrasound (FoCUS)—has revolutionized bedside cardiovascular assessment. FoCUS allows rapid, targeted evaluation of cardiac chambers, valves, intravascular volume, and the pericardium (7, 8). Its clinical utility extends beyond cardiovascular emergencies and has also been described in endocrine-related critical conditions such as pheochromocytoma (9). With adequate training, this technique enhances the physical examination, supports early recognition of life-threatening conditions, and contributes to more rapid and effective therapeutic decision-making (7–10). We report a case of cardiac tamponade as the initial presentation of severe hypothyroidism (myxedema), with the objective of highlighting the diagnostic value of early FoCUS in recognizing tamponade physiology and expediting life-saving management.

## 2 Case presentation

A 62-year-old man with no significant past medical history presented with a six-month history of progressive lower-limb edema and exertional dyspnea. Over the two weeks before admission, his symptoms worsened to dyspnea at rest with generalized anasarca. No precipitating factors were identified.

On presentation to the emergency department, vital signs were blood pressure 122/87 mm Hg, heart rate 112 bpm, respiratory rate 24 breaths/min, and oxygen saturation 90% on room air. He was alert and oriented but exhibited bradyphrenia without focal neurological deficits.

Physical examination revealed myxedematous facies, periorbital edema, and madarosis. Jugular venous distension was evident, and cardiac auscultation demonstrated muffled heart sounds. Pulsus paradoxus was not formally assessed. Additional findings included gynecomastia, abdominal wall edema, and bilateral grade III pitting edema of the lower limbs. There was no previous diagnosis of hypothyroidism; however, clinical examination revealed features suggestive of thyroid dysfunction, and subsequent testing confirmed severe primary hypothyroidism.

**Figure 1:**
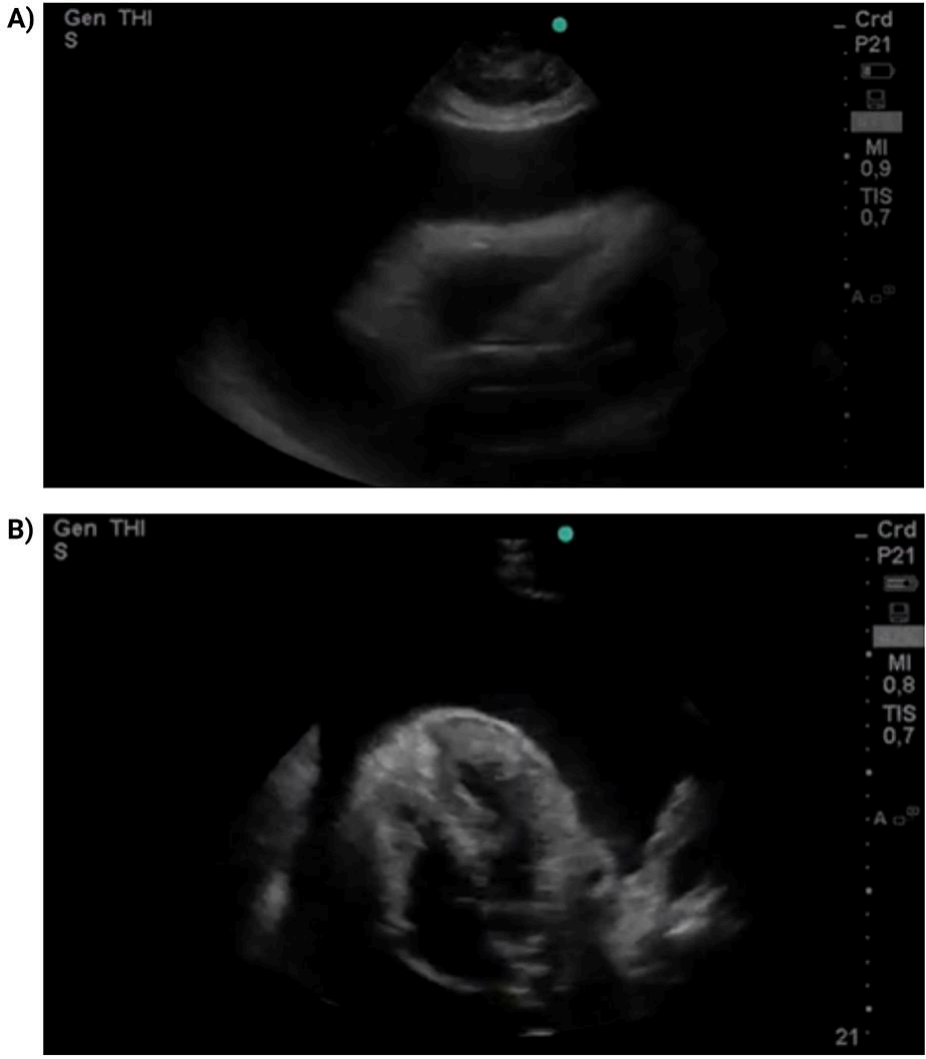
FoCUS parasternal long-axis view **(A)** and subxiphoid four-chamber view **(B)** A massive circumferential pericardial effusion is observed (28 mm in diastole), with collapse of right-sided chambers.

Given the clinical suspicion of cardiac tamponade, focused cardiac ultrasound (FoCUS) was performed in the resuscitation area using a portable *Mindray Z5* ultrasound system with a phased-array transducer. The examination was conducted at the bedside by an emergency physician trained in point-of-care ultrasonography. It showed a massive circumferential pericardial effusion measuring approximately 28 mm in diastole, with right atrial systolic and right ventricular diastolic collapse, consistent with tamponade physiology (Figure 1; Supplementary Videos S1–S2). Interventional cardiology was urgently consulted, and emergency pericardiocentesis was performed via a subxiphoid approach. A total of 1,200 mL of serous fluid was drained, and a Hemovac catheter was left *in situ* for 24 h. The patient experienced immediate relief of dyspnea following the procedure.

Chest radiography obtained during the initial diagnostic evaluation, prior to pericardiocentesis, demonstrated cardiomegaly with a classic “water-bottle” configuration. Pericardial fluid analysis was negative for fungi, acid-fast bacilli, bacterial growth, and malignant cells. Laboratory evaluation (Table 1) showed normocytic anemia (hemoglobin 10.1 g/dL), a monocyte count within the reference range, attributed to a physiological stress response secondary pericardial disease, elevated creatinine (1.8 mg/dL), BUN (34 mg/dL), and pro-BNP (212 pg/mL). Thyroid function tests revealed a markedly elevated thyroid-stimulating hormone (TSH) of 225 mIU/L and suppressed free thyroxine (T4) < 0.07 ng/dL, consistent with severe primary hypothyroidism. In the absence of fever, leukocytosis, or clinical features suggestive of infection or systemic autoimmune disease, an extended infectious or autoimmune workup was not pursued. Serum albumin levels were within the normal range.

**Table 1:** Results of paraclinical tests and pericardial fluid analysis.

Test (unit)	Result	Reference values
Hemoglobin (g/dL)	10.1	13.5–17.2
MCV (fL)	81.5	80–100
White blood cell count, WBC (×10^3^/µL)	7.2	4.0–11.0
Absolute neutrophils (×10^3^/µL)	4.5	1.5–7.0
Absolute lymphocytes (×10^3^/µL)	2.0	1.0–4.0
Absolute monocytes (×10^3^/µL)	0.5	0.1–0.8
Absolute eosinophils (×10^3^/µL)	0.2	0.05–0.5
Platelets (×10⁹/L)	220	150–450
AST (U/L)	28	10–40
ALT (U/L)	25	7–56
Creatinine (mg/dL)	1.8	0.5–0.95
BUN (mg/dL)	34	6–20
Sodium (mmol/L)	132	135–145
Potassium (mmol/L)	4.2	3.5–5.0
Chloride (mmol/L)	103	98–107
Calcium (mg/dL)	9.2	8.5–10.5
Albumin (g/dL)	3.5	3.5–5.0
pro-BNP (pg/mL)	212	<125
TSH (mIU/L)	225	0.37–4.7
Free T4 (ng/dL)	<0.07	0.9–2.3
Pericardial fluid: Direct fungal examination (KOH)	Negative	–
Pericardial fluid: Acid-fast bacilli smear	Negative	–
Pericardial fluid: Bacterial culture	No growth	–
Pericardial fluid: Cytology	Negative for malignant cells	–

MCV, mean corpuscular volume; WBC, white blood cell count; AST, aspartate aminotransferase; ALT, alanine aminotransferase; BUN, blood urea nitrogen; pro-BNP, N-terminal pro–B-type natriuretic peptide; TSH, thyroid-stimulating hormone; free T4, free thyroxine; KOH, potassium hydroxide.

A diagnosis of cardiac tamponade secondary to hypothyroidism was established. The patient was admitted to the intensive care unit and received intravenous hydrocortisone 100 mg every 8 h for 7 days as prophylaxis against adrenal crisis. Serum cortisol and adrenocorticotropic hormone (ACTH) levels were not obtained, which represents a limitation of this case. Levothyroxine was initiated orally with a loading regimen of 200 mcg/day for the first three days, given the unavailability of intravenous formulations in Colombia. The dose was subsequently tapered according to clinical response, targeting a standard replacement of 1.6 mcg/kg/day at discharge. Considering the increased risk of arrhythmias with rapid thyroid hormone repletion, continuous monitoring was implemented. Serial ECGs at 24 and 48 h showed no acute abnormalities, and treatment was well tolerated.

By hospital Day 3, jugular venous distension had resolved, heart sounds regained normal intensity, and oxygen saturation normalized without tachypnea. The patient remained clinically stable with no recurrence of effusion. On Day 8, he was discharged on oral levothyroxine at a standard replacement dose of 1.6 mcg/kg/day and referred for endocrinology follow-up. Outpatient monitoring confirmed normalization of thyroid function on a maintenance dose of 88 mcg/day, with no recurrence of pericardial effusion to date.

**Figure 2:**
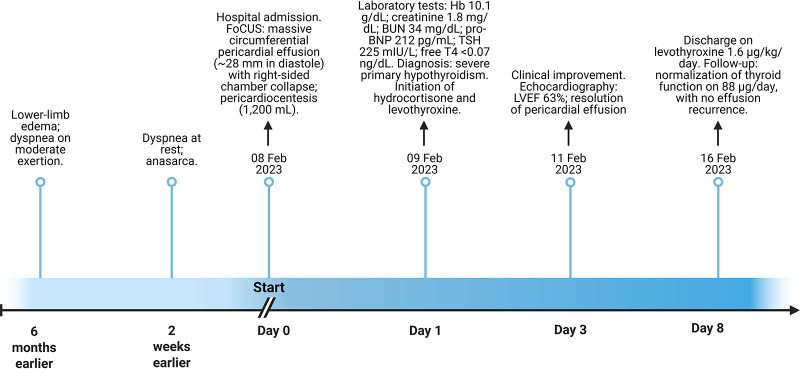
Clinical timeline summarizing presentation, diagnostic findings, and management. FoCUS, focused cardiac ultrasound; LVEF, left ventricular ejection fraction; TSH, thyroid-stimulating hormone; free T4, free thyroxine; BUN, blood urea nitrogen; Hb, hemoglobin.

A detailed timeline summarizing the clinical course, diagnostic findings, and therapeutic interventions is presented in Figure 2.

## 3 Discussion

Although FoCUS is an established bedside tool, this report highlights its pragmatic value in promptly recognizing tamponade physiology in a rare endocrine etiology (severe hypothyroidism) with nonspecific clinical findings, particularly in resource-limited settings.

Pericardial effusion is a well-recognized complication of hypothyroidism; however, progression to cardiac tamponade is exceptionally rare. Fewer than 3% of patients with mild hypothyroidism develop tamponade, whereas the risk rises markedly in myxedema crisis, with rates reported as high as 80% (10–12). Proposed mechanisms include increased capillary permeability, impaired lymphatic drainage, and leakage of protein-rich fluid—particularly albumin—into the pericardial space (12, 13).

FoCUS was performed at the bedside using a portable point-of-care system with a phased-array transducer (14). Standard views (parasternal long- and short-axis, apical four-chamber, and subcostal) demonstrated hallmark features of tamponade physiology, including right atrial systolic and right ventricular diastolic collapse. Although FoCUS does not replace comprehensive echocardiography for detailed hemodynamic assessment, it provides rapid, actionable information in time-critical settings. Its main limitations include operator dependency and reduced image quality in suboptimal acoustic windows; nevertheless, when performed by trained clinicians, FoCUS constitutes a valuable extension of the physical examination in acute cardiovascular care (14).

The hemodynamic impact of an effusion depends not only on absolute volume but also on the rate of accumulation. Large, slowly evolving effusions may be relatively well tolerated, while smaller but rapidly accumulating collections can precipitate cardiovascular collapse (15). This dynamic explains the broad clinical spectrum and underscores the difficulty of anticipating which patients will deteriorate to tamponade.

In this context, FoCUS provides rapid, bedside recognition of key echocardiographic features of tamponade—right atrial systolic and right ventricular diastolic collapse, and inferior vena cava plethora—thereby narrowing diagnostic uncertainty and guiding time-critical decisions (16–19). Unlike formal echocardiography, FoCUS can be performed within minutes in the emergency department and used as an extension of the physical examination, especially when classical signs such as Beck's triad are absent or incomplete (20, 21). Consistent with this expanded role, ECG–FoCUS integration has demonstrated diagnostic and triage benefits beyond tamponade scenarios. In a recent report from our group, the combined bedside approach enabled early recognition of Wellens’ syndrome complicated by acute heart failure and timely reperfusion, underscoring its adaptability in resource-limited settings (22).

In our patient, FoCUS immediately demonstrated a massive circumferential effusion with right-sided chamber collapse, expediting pericardiocentesis with drainage of 1,200 mL of serous fluid and prompt clinical improvement. Subsequent biochemical testing confirmed severe primary hypothyroidism as the underlying etiology. This favorable course highlights the value of integrating clinical suspicion, targeted ultrasound, and thyroid function testing in endocrine-related cardiovascular emergencies.

Literature supports this approach. Othon-Martínez et al. described timely POCUS-guided recognition of tamponade enabling urgent intervention (23). Khaw and Chin reported hypothyroidism-associated effusion after radioactive iodine therapy that resolved with thyroid hormone replacement alone, without drainage (24). In contrast, Patil et al. documented primary hypothyroidism with tamponade requiring both pericardiocentesis and levothyroxine (25). Beyond single cases, Wang et al. emphasized in a larger cohort the need to integrate clinical, echocardiographic, and biochemical data for accurate diagnosis and management (13).

Key lessons from this case are threefold, hypothyroidism should remain in the differential diagnosis of large or unexplained pericardial effusions, particularly when clinical features are nonspecific (10–13). In addition, FoCUS should be embedded in emergency and critical-care workflows as a direct extension of the physical exam to accelerate recognition of tamponade physiology and trigger definitive management. Finally, timely pericardiocentesis, when indicated, together with appropriate thyroid hormone replacement, can reverse hemodynamic compromise and reduce the risk of recurrence, consistent with prior reports**.**

## 4 Conclusions

This case underscores the clinical value of FoCUS as a rapid, reliable bedside tool for the early identification of pericardial effusion and cardiac tamponade. Because clinical presentation is often nonspecific and a complete Beck's triad is frequently absent, physical examination alone may be insufficient to establish the diagnosis. In this patient, FoCUS promptly demonstrated tamponade physiology, expediting pericardiocentesis and directly informing acute management. FoCUS should be integrated into routine emergency and critical-care practice as an extension of the physical examination, particularly in patients with unexplained cardiomegaly or hemodynamic compromise. When combined with clinical assessment, FoCUS supports early, targeted interventions and can be lifesaving.

## Data Availability

The original contributions presented in the study are included in the article/Supplementary Material, further inquiries can be directed to the corresponding author.
